# The role of social support in moderating the impact of cumulative trauma on suicidal thoughts and behaviors

**DOI:** 10.3389/frcha.2025.1677475

**Published:** 2025-12-09

**Authors:** Martine Hébert, Amélie Tremblay-Perreault, Laetitia Mélissande Amédée

**Affiliations:** 1 Département de Sexologie, Université du Québec à Montréal, Montréal, QC, Canada; 2 Department of Psychiatry and Behavioural Neurosciences, Offord Centre for Child Studies, McMaster University, Hamilton, ON, Canada

**Keywords:** suicidality, social support, cumulative trauma, suicidal ideations, suicidal attempts

## Abstract

**Introduction:**

Suicidality in adolescence is a significant public health concern. One potential correlate of suicidality is exposure to childhood trauma. Research shows that cumulative trauma is associated with a higher risk of suicidality during adolescence, with a dose-response pattern between the number of adversities and suicidal outcomes. However, not all adolescents exposed to adversity report suicidal thoughts or behaviors, suggesting that protective factors, such as social support, may buffer this association. This cross-sectional study examined gender differences in the prevalence of suicidal thoughts and behaviors, cumulative trauma, and social support among high school students. The study also sought to determine whether cumulative trauma was related to an increased likelihood of suicidal thoughts and behaviors, and whether social support moderated the association.

**Methods:**

Data were collected from a representative sample of 8,194 adolescents in Grades 9–11 in Quebec, Canada.

**Results:**

Girls were more likely than boys to report both suicidal ideation and attempts. The number of traumas experienced was positively associated with the prevalence of suicidal ideation and attempts, with each additional trauma linked to an estimated 65%–80% increase in odds, indicating a large dose-response association. After controlling for sociodemographic characteristics, social support was associated with an attenuation of the relationship between cumulative trauma and suicidal ideation. For suicide attempts, social support showed a direct protective effect among adolescent girls, but this association was not observed among boys.

**Discussion:**

These findings highlight the relevance of early identification and support services for youth reporting multiple adversities and lower social support.

## 1 Introduction

Suicidal thoughts and behaviors (STB) among adolescents represent a major public health concern. Adolescence is a critical developmental period marked by increased vulnerability to mental health difficulties, including suicidality, which is one of the leading causes of death in this age group (1). Interpersonal violence experienced in the home environment, including sexual abuse, physical abuse, emotional abuse, and exposure to interparental violence, has been consistently linked to poor mental health outcomes and suicidality (2, 3). Youth are also exposed to interpersonal trauma outside their home, such as peer victimization, which may magnify the effects of child maltreatment on STB (4). In a systematic review and meta-analysis of longitudinal studies, child maltreatment and bullying were found among the most potent risk factors for suicide attempts for adolescents and young adults (5).

Research shows that exposure to multiple forms of childhood trauma significantly increases the risk of suicidality during adolescence, following a dose-response relationship between the number of adversities and the risk of STB (6). However, not all adolescents exposed to interpersonal trauma will display STB, suggesting that protective factors may play a crucial moderating role (7). The present study aimed to investigate the relationship between cumulative trauma and STB among adolescents while assessing the buffering effect of one of these factors, namely social support.

Social support has long been conceptualized as having a protective influence on well-being. Several hypotheses have been proposed to explain the mechanism underlying this effect, with two prominent models. The first, the main-effect model, suggests that social support exerts a general positive influence on one's psychosocial adaptation, independent of stress exposure. The second, the buffering effect model, proposes that social support functions as a protective factor, especially in the context of stress (8, 9).

A synthesis of 60 meta-analyses revealed a strong association between social support and psychological adjustment across various outcomes and age groups (10). Having multiple and varied sources of social support (e.g., family members, peers, and other significant adults) can enhance resilience and mitigate the risk of STB (11, 12). In the face of adversity, social support from diverse sources may provide emotional validation, coping resources, and a sense of belonging, which are essential for psychological well-being (13, 14). Data suggest that adolescents with strong social support are less likely to engage in suicidal behaviors, even when exposed to significant adversity (7).

Despite consistent evidence supporting the protective role of social support in adolescents' overall well-being, research examining the association between cumulative trauma and STB remains limited. Moreover, in the few studies that did explore this question, authors mostly focused on social support offered at school by teachers and peers (7), disregarding the potential contribution of support provided by parents, siblings, significant adults other than teachers, and friends outside of school. Yet, in adolescence, parents and siblings remain important sources of support and can act as protective figures against the development of psychopathology (15). Examining the combined effect of these various sources of support will offer a more comprehensive understanding of their potentially buffering effect in the context of cumulative trauma.

Moreover, although evidence points to gender differences in STB and in related protective factors during adolescence (4, 5), most studies examining the association between social support, cumulative trauma, and STB have not conducted gender-stratified analyses. Research consistently reports gender-specific STB patterns among adolescents. For instance, a meta-analysis identified interpersonal problems as a risk factor for STB in girls but not in boys (5), suggesting that protective factors may differ by gender (4). Thus, further gender-specific analyses are needed to clarify the potential moderating role of social support in the relationship between childhood trauma and STB.

Given growing concerns about adolescent suicidality, this study had three main objectives. First, it examined the prevalence of STB, cumulative trauma, and social support among high school students, while exploring potential gender differences. Second, it investigated the association between cumulative trauma and STB, testing whether greater trauma exposure increased the likelihood of STB. Third, drawing on the buffering effect model, it assessed whether social support moderated the link between cumulative trauma and suicidality, with higher support expected to weaken this association.

This study addresses gaps in the literature by adopting a comprehensive conceptualization of both cumulative trauma and social support. Multiple trauma forms were included, namely emotional, physical, and sexual abuse, as well as in-person and online peer victimization. Social support was assessed across several sources: parents, siblings, significant adults other than parents, and friends. Given prior evidence of gender-specific STB patterns, analyses were conducted separately for boys and girls.

## 2 Methods

### 2.1 Participants

This cross-sectional study used data from the Youth Romantic Relationships Survey, which aimed to assess the prevalence of interpersonal violence among high school students in Quebec, Canada. Using a one-stage stratified cluster sampling, 34 schools were randomly selected from a list of schools provided by the Ministry of Education. We used eight strata to categorize schools based on metropolitan geographical area, school status (public or private), teaching language (French or English), and socioeconomic deprivation index. Data were collected through an anonymous, self-administered online questionnaire completed during class time under the supervision of trained research staff. This procedure minimized social desirability bias and ensured confidentiality. Although retrospective self-report may be subject to recall bias, standardized and behaviorally specific items were used to improve accuracy in reporting traumatic experiences and suicidal thoughts or behaviors. Class and overall student response rates were calculated as the proportion of students who provided written consent relative to the number of students invited to participate. In most classes (320 of 329), all solicited students participated (100% response rate). For the remaining classes, participation ranged from 90% to 98%. Overall, 99% of solicited students agreed to take part in the study. The ethics institutional review board of the affiliated university approved this study. The initial sample consisted of 8,230 youth; 36 were excluded from the study for invalid responses or missing data on all study variables. A sample weight was applied to adjust for the nonproportionality of the school sample relative to the target population, ensuring that estimates were representative of the Quebec high school population (Grades 10–12; effective sample size: *N* = 6,531 participants; 57.8% girls, *M*_age_ = 15.35).

### 2.2 Measures

#### 2.2.1 Suicidal thoughts and behaviors

Participants answered two dichotomous questions (0—no, 1—yes). To assess suicidal ideation, this study used an item from the National Longitudinal Survey of Children and Youth (“Have you ever seriously thought of committing suicide?”; 16). To measure suicidal attempts, participants were asked the following question: “Have you ever attempted suicide?”.

#### 2.2.2 Cumulative trauma

A composite score of cumulative trauma was computed by summing dichotomous scores of five forms of interpersonal violence, namely sexual abuse, physical abuse, emotional abuse, exposure to interparental violence, and peer victimization.

Child sexual abuse was assessed through two items adapted from Finkelhor et al. (17): “Have you ever been touched sexually when you did not want to, or have you ever been manipulated, blackmailed, or physically forced to touch sexually?”, and “Has anyone ever used manipulation, blackmail, or physical force, to force or obligate you to have sex [including all sexual activities involving oral, vaginal, or anal penetration]?”). Adolescents answered to two items from the *Inventory of Parent and Peer Attachment* (18) regarding their experiences of emotional abuse (for eg., “My mother tells me hurtful things and/or insulting things”). Two items from the *Early Trauma Inventory Self-Report–Short Form* (19) (e.g., “Have you ever been physically hit by a member of your family?”) were used to report experiences of physical abuse. For each of the aforementioned forms of maltreatment, participants who endorsed at least one of the two situations were considered as having experienced this particular form of maltreatment.

An adapted version of the *Revised Conflict Tactics Scales* (CTS2; 20) was used to assess lifetime prevalence of exposure to interparental violence. Participants were asked to rate the frequency of exposure to eight different manifestations of psychological and physical violence occurring between parents. If they witnessed any manifestation at least once, they were considered to have been exposed to interparental violence. Three items were drawn and adapted from Statistics Canada (16): “somebody made you feel excluded or left out” and “somebody harassed you using electronic technologies”) and Chamberland et al. (21): “somebody harassed you at school or somewhere else”) to measure the overall prevalence in the last 12 months of peer victimization. A dichotomous score was created to distinguish participants who reported at least one experience of peer victimization (1) from participants who did not (0).

#### 2.2.3 General social support

The item “Do you think the following people would really listen to you and help you feel better if you really needed it?” was drawn from the *Enquête sociale et de santé auprès des enfants et des adolescents 1999* (22) to assess social support. Adolescents were asked to report to what extent (0—Not at all; 1—A little or A lot) they believe they could count on one of their parents, another significant adult, a brother or sister, and a friend. A total score was computed by adding scores for all four sources of support to yield a score of 0–4. A higher score indicates a wider network of social support.

#### 2.2.4 Sociodemographic characteristics

Participant's sociodemographic characteristics were collected, namely: age, gender, family structure (0—living with two parents; 1—living in a single-parent family, or any other arrangement), maternal level of education (0—post-secondary; 1—elementary or high school), religious affiliation (0—does not identify with any religion; 1- identifies with a religion), sexual orientation (1—sexual minority or 0—not), and immigration status of parents (0—both parents born in Canada; 1—at least one parent born outside of Canada).

### 2.3 Data analysis

Descriptive statistics of the variable are provided in Table 1. Both suicidal outcomes and interpersonal violence variables were operationalized as dichotomous indicators (yes/no) to align with national surveillance standards and prior epidemiological studies. This approach facilitates comparability across violence types, supports computation of a cumulative trauma index, and reduces distortion from low-frequency events. In contrast, social support was treated as a continuous count of distinct supportive sources to preserve variability and capture the breadth of adolescents' support networks.

**Table 1:** Descriptive statistics of variables.

Variables	*M*	*SE*	%
Suicidal ideation (yes)			26.4
Suicidal attempts (yes)			9.0
Social support (range 0–4)	3.20	0.03	
Cumulative trauma (range 0–5)	2.06	0.05	
Sexual abuse (yes)			10.3
Emotional abuse (yes)			42.3
Physical abuse (yes)			25.4
Exposure to interparental violence (yes)			60.3
Peer victimization (yes)			71.0
Covariates			
Age	15.35	0.11	
Single-parent family (yes)			24.1
Low level of education of mother (yes)			29.3
Immigrant parents (yes)			73.3
Religious affiliation (yes)			70.2
Sexual minority status (yes)			17.4

To account for the stratified cluster sampling of the data, the complex sample option of the SPSS 27 software was used to conduct the analyses. Analyses were conducted using the Complex Sample module in SPSS, which applies pairwise deletion for missing data. We had less than 10% of missing data for most study variables except for mother's level of education (14.2%), peer victimization (10.7%), and cumulative trauma (15.4%). Chi-square analyses revealed that missingness was related to variables included in the dataset, and therefore was deemed Missing at Random (MAR). As recommended by Bell et al. (23), listwise or pairwise deletion is acceptable in the context of complex sample designs when data are assumed to be MAR.

For the first objective, *t*-test analyses were conducted to compare levels of social support and the average number of traumas experienced between boys and girls. Descriptive statistics were used to obtain the prevalence of both suicidal ideation and suicide attempts for the full sample as a function of the number of traumas reported.

For the second and third objectives, two separate logistic regressions were tested to examine the effects of social support and cumulative trauma on, first, suicidal ideation, and second, suicide attempts. First, control variables (age, family composition, mother's level of education, immigrant status, religious affiliation, and sexual minority status) were identified through chi-square and *t*-tests comparing adolescents with and without any report of STB on multiple socio-demographic characteristics. Covariates were entered in the first step, and cumulative trauma, social support, and the cumulative trauma × social support interaction term in the second. To facilitate interpretation, social support was mean-centered. Analyses were tested separately for boys, girls, and the full sample. Given empirical evidence of gender specific patterns of STB (5), subgroup analyses were conducted to explore potential differences in the strength of associations. Building upon the buffering effect model (8), interaction terms were added to examine whether the relationship between cumulative trauma and suicidality varied as a function of social support. *post-hoc* analyses were conducted to interpret the significant interaction effects. We fitted additional fully adjusted multivariate logistic regression models stratified by levels of support to examine the associations between cumulative trauma and STB. Social support was categorized to reflect low (<-1SD from the mean), and moderate to high levels (-1SD and over).

## 3 Results

### 3.1 Descriptive statistics

**Figure 1:**
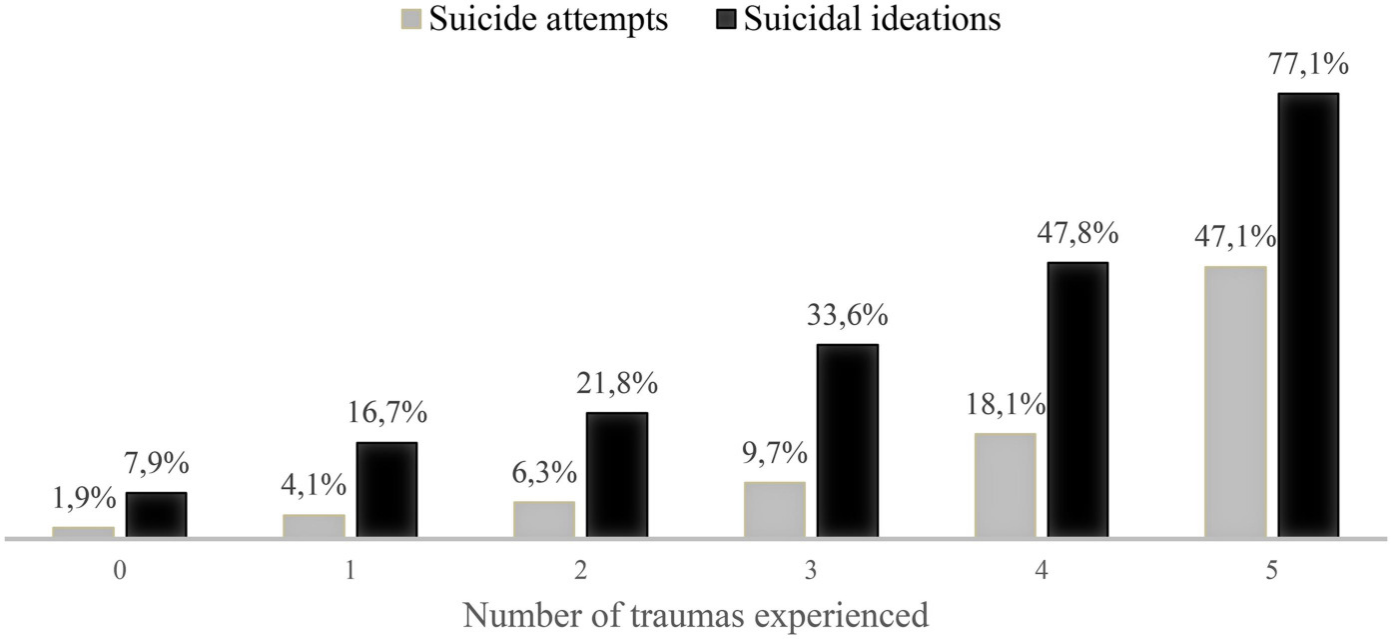
Percentage of adolescents reporting suicidal thoughts and behaviors as a function of the number of traumas experienced.

Results of a chi-square analysis indicate that girls (31%) were more likely than boys (19.9%) to have experienced suicidal ideation [*χ*^2^(1, *N* = 4,726.03) = 124.03, *p* < 0.001]; the prevalence in the full sample was 26.4%. Nine percent of the whole sample reported having attempted suicide at least once. Again, the prevalence was higher in girls (11.9%) than in boys [4.9%; *χ*^2^(1, *N* = 5,786.16) = 117.65, *p* < .001]. Boys (*M* = 3.57, *SE* = 0.2) reported significantly more sources of support [*t*(26) = −2.63; *p* = .01] than girls (*M* = 3.52, *SE* = 0.2). Girls (*M* = 2.21, *SE* = 0.4) reported more trauma experiences [*t*(26) = 10.73; *p* < .01] than boys (*M* = 1.63, *SE* = 0.4). In Figure 1, the prevalence of teenagers reporting suicidal ideations and attempts is illustrated as a function of the number of different forms of trauma experienced.

### 3.2 Moderating role of social support

Table 2 displays the results of the logistic regressions for suicidal ideation for the full sample, for boys, and for girls. For all models that were tested, a greater number of experienced traumas was associated with an increased likelihood of reporting suicidal ideation. Cumulative trauma showed a large effect on suicidal ideation. Odds ratios between 1.68 and 1.73 indicate that each additional trauma type was associated with approximately a 70% increase in the odds of suicidal ideation for girls and boys. For boys and for girls, social support interacted significantly with cumulative trauma.

**Table 2:** Results of the logistic regression evaluating the interaction effects between cumulative trauma and social support for suicidal ideation.

Variables	Girls			Boys			Full sample		
	OR	95% CI	*p* value	OR	95% CI	*p* value	OR	95% CI	*p* value
Covariates									
Age	1.04	[.93–1.18]	.475	1.13	[1.02–1.25]	.020	1.07	[.97–1.14]	.167
Single-parent family	1.53	[1.26–1.85]	<.001	1.38	[1.02–1.87]	.036	1.48	[1.29–1.70]	<.001
Education level of mother	1.29	[1.08–1.53]	.007	1.04	[.76–1.44]	.797	1.19	[1.04–1.37]	.014
Immigrant parents	1.25	[1.02–1.52]	.029	1.46	[1.07–1.99]	.020	1.29	[1.06–1.57]	.013
Religious affiliation	.72	[.56–.92]	.011	.70	[.52 -.92]	.017	.73	[.61 -.87]	.001
Sexual minority status	1.73	[1.32–2.27]	<.001	1.73	[1.27–2.36]	.001	1.81	[1.49–2.19]	<.001
Cumulative trauma	1.68	[1.55–1.82]	<.001	1.72	[1.56–1.90]	<.001	1.73	[1.61–1.85]	<.001
Social support	1.13	[.83–1.52]	.426	.93	[.72–1.21]	.565	1.04	[.84–1.28]	.735
Cumulative trauma × Social support	.89	[.81 -.97]	.011	.92	[.85–1.00]	.043	.91	[.85 -.97]	.006

OR, Odds ratio; CI, Confidence Interval.

Table 3 presents the *post-hoc* results of the logistic regressions regarding the relationship between cumulative trauma and the risk of suicidal ideation for varying levels of social support. For adolescents reporting low social support, the association between cumulative trauma and suicidal ideation was large (girls: OR = 2.15; boys: OR = 1.95), indicating that each additional trauma type more than doubled the odds of suicidal ideation in girls and nearly doubled them in boys. For those with moderate to high social support, the association was notably weaker (girls: OR = 1.59; boys: OR = 1.70). This shift suggests a buffering effect of social support: although trauma exposure continues to predict suicidal ideation, the presence of stronger support systems mitigates its impact to a moderate level.

**Table 3:** *post-hoc* logistic regression associations between cumulative trauma and suicidal ideation as a function of levels of social support.

Adjusted^a^ Multivariate Models	Girls			Boys			Full sample		
	OR	95% CI	*p* value	OR	95% CI	*p* value	OR	95% CI	*p* value
Low social support									
Cumulative trauma (0–5)	2.15	[1.82–2.53]	<.001	1.95	[1.54–2.47]	<.001	2.09	[1.81–2.42]	<.001
High-moderate social support^b^									
Cumulative trauma (0–5)	1.59	[1.47–1.72]	<.001	1.70	[1.56–1.84]	<.001	1.66	[1.56–1.77]	<.001

OR, Odds ratio; CI, Confidence Interval.

a Adjusting for age, family configuration, mother's level of education, immigrant parent status, religious affiliation, sexual minority status.

b Moderate and high levels of social support were combined as no participants had scores superior to one standard deviation from the group mean.

Results for suicide attempts are displayed in Table 4. Cumulative trauma exhibited a strong main effect on suicide attempts for both girls (OR = 1.78), boys (OR = 1.64), and the full sample (OR = 1.82), indicating that each additional trauma type increased the odds of a suicide attempt by roughly 65%–80%. This represents a large effect size. Social support demonstrated a main effect only among girls (OR = 0.67), indicating a moderate protective influence; each one-unit increase in social support reduced the odds of a suicide attempt by approximately 33%. This effect was not observed among boys or in the whole sample. The interaction between cumulative trauma and social support was nonsignificant in all models, suggesting that social support did not moderate the association between cumulative trauma and suicide attempts.

**Table 4:** Results of the logistic regression evaluating the interaction effect between cumulative trauma and social support for suicide attempts.

Variables	Girls			Boys			Full sample		
	OR	95% CI	p value	OR	95% CI	*p* value	OR	95% CI	*p* value
Covariates									
Age	1.00	[.88–1.13]	.936	1.38	[1.14–1.66]	.002	1.08	[.96–1.21]	.188
Single-parent family	1.56	[1.03–2.36]	.037	.91	[.46–1.79]	.775	1.40	[.99–1.96]	.054
Education level of mother	1.65	[1.28–2.13]	<.001	1.57	[1.06–2.32]	.026	1.62	[1.30–2.03]	<.001
Immigrant parents	1.13	[.80–1.59]	.468	.86	[.52–1.43]	.538	1.04	[.75–1.45]	.805
Religious affiliation	.69	[.51–.92]	.014	.62	[.42–.93]	.022	.69	[.55–.86]	.002
Sexual minority status	1.93	[1.51–2.48]	<.001	3.06	[1.89–4.96]	<.001	2.29	[1.84–2.85]	<.001
Cumulative trauma	1.78	[1.51–2.10]	<.001	1.64	[1.39–1.95]	<.001	1.82	[1.60–2.07]	<.001
Social support	.67	[.48–.94]	.021	1.13	[.60–2.11]	.699	.80	[.63–1.02]	.066
Cumulative trauma × Social support	1.06	[.94–1.18]	.341	.85	[.68–1.06]	.143	1.00	[.92–1.09]	.953

OR, Odds ratio; CI, Confidence Interval.

## 4 Discussion

The present study examined associations between cumulative trauma and STB among adolescents, as well as the moderating role of social support, using a cross-sectional design. Consistent with past studies, the prevalence of both suicidal ideation and attempts was notably higher among girls than boys, aligning with prior research on gender differences in suicidality (4, 5). Cumulative trauma was strongly associated with an increased likelihood of both suicidal ideation and suicide attempts, which is consistent with studies that have documented a graded dose response relationship between childhood adversities and impaired psychological and physical health (6, 24).

Our results did not reveal a significant main effect of social support on suicidal ideation for either girls or boys, but social support significantly moderated the association between cumulative trauma and suicidal ideation. These results provide support for the stress buffering hypothesis (8, 9). Adolescents with a broader support network reported a reduced likelihood of suicidal ideation despite high levels of interpersonal trauma, suggesting the value of fostering positive social environments could be particularly beneficial for adolescents who have experienced multiple forms of interpersonal trauma, helping to mitigate the heightened risk of suicidal ideation. Our findings regarding suicidal ideation are consistent with those of Mackin et al. (25), who also found a buffering effect of parental and peer support on suicidal ideation among adolescent girls experiencing interpersonal stressors such as relationship breakups, peer victimization, and sexual violence. Similarly, Forster and colleagues (7) reported that teacher and peer support buffered the association between adverse childhood experiences and suicidal ideation among high-school students.

However, no significant interaction was found for suicide attempts, implying that the association between cumulative trauma and risk of suicide attempts is not influenced by levels of social support. Social support appears to have a direct protective effect against suicide attempts in adolescent girls, indicating that it provides significant benefits, regardless of whether they have a history of cumulative trauma. This pattern is more consistent with the main effect model, in which social support contributes directly to one's psychosocial adaptation, regardless of stress exposure. However, this protective effect was not observed for boys. Once adolescent boys reach the stage of attempting suicide, the protective benefits of social support may not be evident, particularly if other risk factors such as conduct problems are present (5).

Taken together, our results contrast with a study conducted among Chinese adolescents that examined the associations between adverse childhood events, social support, and STB (12). First, Wan and colleagues reported a main effect of social support on both suicidal ideation and suicide attempts. Second, they found no moderating effect for suicidal ideation and found an interaction effect for suicide attempts for boys but not for girls. Several factors may account for these discrepancies. For instance, the conceptualization of adverse childhood events did not include peer victimization, which is a particularly salient stressor during adolescence. In addition, cultural differences may play a role; in a more collectivistic culture, such as China, social support may be a more normative and expected resource and thus operate primarily as a direct protective factor (12, 26). Whereas, in more individualistic contexts such as Quebec, access to strong support networks may vary more between adolescents and function more as a buffering resource in times of stress (26, 27).

The findings of this study have several implications for suicide prevention and intervention strategies. Given the link between cumulative trauma and suicidality, early identification and support for trauma-exposed youth should be a public health priority. Schools, healthcare providers, and community organizations must implement trauma-informed care approaches that recognize the impact of past victimization on adolescent mental health. Moreover, the protective role of social support for suicidal ideation emphasizes the importance of strengthening family and peer relationships. Programs that promote parental engagement and peer mentorship could be valuable in mitigating the effects of trauma.

This study drew on a large, stratified, and representative sample of high school students in Quebec, strengthening the generalizability of the findings. Rather than focusing on isolated forms of interpersonal trauma, it examined the cumulative impact of multiple forms of interpersonal trauma, providing a more comprehensive understanding of risk factors for STB.

Nonetheless, the results should be interpreted in light of some limitations. First, the cross-sectional design precludes causal inference and prevents asserting that exposure to risk and protective factors preceded suicidal ideation and attempts. As data came from a larger research project, some limitations relate to the available measures. For instance, trauma types in the cumulative trauma exposure score were assessed over different time frames; reports of CSA were lifetime, whereas peer victimization covered the past year. Cumulative trauma rates may have been underestimated, potentially biasing results. Moreover, the trauma list was not exhaustive, with parental neglect, among others, unassessed. Self-report data may be affected by recall bias, particularly for retrospective accounts of victimization and sensitive outcomes such as suicide attempts. Finally, findings should also be interpreted in light of contextual boundaries: the study was conducted in Québec, where sociocultural and service-system factors may differ from other regions, limiting generalizability. Future research using longitudinal designs is needed to clarify causal mechanisms and to examine whether the observed patterns hold across cultural contexts. Finally, data on socioeconomic indicators such as family income were not available, limiting the ability to account directly for social class disparities. Although proxy measures (maternal education, family structure, parental immigration status) were included, future studies should incorporate comprehensive indicators of socioeconomic status to better capture its role in suicidal risk processes.

## 5 Conclusion

This study highlights the association between cumulative trauma and adolescent suicidality, as well as the important role of social support in attenuating suicidal ideation. Future research and policy initiatives should aim to strengthen trauma-informed care, reinforce protective support systems, and advance suicide prevention strategies tailored to youth exposed to multiple adversities.

## Data Availability

The data that support the findings of this study are available from the corresponding author upon reasonable request. Requests to access the datasets should be directed to Martine Hébert, hebert.m@uqam.ca.
